# Supplementary material for: Mixed methods study evaluating the implementation of the WHO hand hygiene strategy focusing on alcohol based handrub and training among health care workers in Faranah, Guinea
Source: PLoS One. 2021 Aug 26;16(8):e0256760. doi: 10.1371/journal.pone.0256760 (PMC8389517; doi:10.1371/journal.pone.0256760)
Supplement: Attachment — Submitted filename: 01_06_rebuttal letter.docx [file pone.0256760.s002.docx]

Response to reviewer’s comment PONE-D-20-12650R1-rejection:

*Reviewer #3: Authors should focus on the 2nd follow-up and on the qualitative data. It seems that the same weight is given to the baseline, first follow-up, second follow-up and qualitative data. It is confusing. Authors say “The design and methods of this study have been described in detail elsewhere [12]’, but this is incomplete – results have also been presented in that publication. That fact is also not acknowledged in the discussion section.*

We are very sorry not to have acknowledged your main suggestion adequately and would like to apologize for this miscommunication and poor presentation on our side. We appreciate your concern and would like to state that we totally agree that double publication would be a major flaw of publication ethics. It is in no way our intention to double publish our results, we therefore clearly and transparently marked all already published sections and state

“The design, methods of the quantitative part and results of baseline and first follow-up of this study have been described in detail elsewhere (Ref.). To show long-term trends, including potential waning of knowledge and compliance, some results of baseline and first follow-up are paraphrased and compared to second follow-up in the discussion section. Previously published results of baseline and first follow-up are clearly marked.” (Page 5)

We readdressed the comment of reviewer #3 and readapted the manuscript focusing on the second follow-up and qualitative data:

- In the results section, we completely removed all baseline and follow-up data. Only differences between results from 2^nd^ follow up and earlier assessments as well as p-values for these comparisons are reported, since these differences and p-values are new findings in their own right.
- In the discussion section, in order to assess the long-term effects of the WHO Hand Hygiene Strategy and to show trends and potential problems of waning compliance or knowledge, we deemed it necessary to include some of the baseline and first follow-up results to compare to the second follow-up. In our opinion, the second follow-up is not meaningful without a reference point and can’t be used to assess long-term effects of the WHO strategy on hand hygiene improvement in the partner hospital of Faranah.
- Figure 1 and 2 compare results with previously published data and are therefore displayed in the discussion section. If this is not deemed appropriate we would propose to move these figures to results section.
