# Supplementary material for: Mixed methods study evaluating the implementation of the WHO hand hygiene strategy focusing on alcohol based handrub and training among health care workers in Faranah, Guinea
Source: PLoS One. 2021 Aug 26;16(8):e0256760. doi: 10.1371/journal.pone.0256760 (PMC8389517; doi:10.1371/journal.pone.0256760)
Supplement: Attachment — Submitted filename: rebuttal letter.docx [file pone.0256760.s003.docx]

Rebuttal letter

PONE-D-21-00332

Mixed methods study evaluating the implementation of the WHO Hand Hygiene Strategy PLOS ONE

General:

- changes made to the financial disclosure have been included in the updated statement in our cover letter
- file naming has been changed following the PLOS ONE's style requirements
- the data availability statement has been addressed in the cover letter
- data not shown: Thank you, we now show summary statistics of the data formerly “not shown”. The data themselves are, as explained above, available on request.

“A sub-analysis of knowledge scores was conducted using only questionnaires from HCWs who had participated in baseline and both follow-ups. This sub-analysis of 22 HCWs showed the same tri-phasic trend with significant increase from baseline (median 14.0; IQR 12.0-15.0) to first follow-up(median 20.5; IQR 19.0-22.0, p<0.001), significant increase from baseline to second follow-up (median 18.0; IQR 16.0-19.0, p<0.001) and significant decrease between the two follow-ups (p=0.002). It was, therefore, decided to include all HCWs in the knowledge analysis (Table 3).”

**Reviewer #1:** Full Title: Reading through, the study is among “health workers” i suggest you include that please. I suggest you including study location too as the full title and short title still have same word count.

- Thank you, the new full title is “Mixed methods study evaluating the implementation of the WHO Hand Hygiene Strategy focusing on local production alcohol based handrub and training among health care workers in Faranah, Guinea” and short title “Mixed methods study evaluating the implementation of the WHO Hand Hygiene Strategy”

Abstract

L12: I suggest including that baseline here

- Thanks for this comment. However, baseline data have been published elsewhere, so we have removed them from this manuscript upon request by an earlier reviewer and the journal’s editor. Instead, we provide the reference in the manuscript where baseline results can be found: Müller et al. 2020 doi: 10.1186/s13756-020-00723-8

L18: I suggest you include the word “of”

- Thank you, we now state: “HCW’s were concerned about overconsuming of ABHR”

Conclusion

L3: This abbreviation has not been defined

- Thank you for spotting this mistake, abbreviation is now defined in L12 “(…) the relaunch of the local production of alcohol-based handrub (ABHR).”

Introduction

L23: Again, this is not defined, if it is referring alcohol-based hand-rub, define it first and continue using the abbreviation throughout.

- Thank you for spotting this mistake, abbreviation is now defined in L10 “(…) unsustainable supply of alcohol-based hand rub (ABHR),”

L24: I suggest you continue using HH since it has be defined or stay with household through the manuscript rather than mixing the abbreviations with the full word.

- Thank you, we now use the acronym HH for hand hygiene throughout the manuscript

Study Settings

L7: I suggest including literature on other sources and access of water in the hospital if there is no water running system.

- We added more details on page 5 L7ff: “When the project started, the hospital had insufficient access to ABHR and water, and no running water at all: water was fetched from a borehole and distributed to the various points of use by buckets.”

Quantitative study

L20: This definition and abbreviation should have come above, as the abbreviation is used in the abstract and L23 in the introduction already.

- Thank you, we introduced the abbreviation in L10 in the introduction.

Qualitative study L11: How many participant per FGDs?

- Thank you, we added “study with 22 in-depth semi-structured interviews (IDIs) and 6 focus group discussions (FGDs) with 4-8 participants each” in p7 L11ff and more detailed information can be found in p9, L 12ff “Moreover, six FGDs were done, each with different professional groups (8 members of the operating theater, 8 nurses, 6 laboratory workers, 6 midwives, 5 doctors and 4 members of the maintenance team).”

Hand Hygiene Knowledge

L13: Typo, the letter "e" is left out

- Thank you, we corrected this typo.

**Reviewer #2:** To be clear, below are the 5 WHO Hand Hygiene (HH) Strategies 1a. System change – alcohol-based handrub at point of care 1b. System change – access to safe, continuous water supply, soap and towels 2. Evaluation and feedback 3. Training and education 4. Reminders in the workplace 5. Institutional safety climate Baseline evaluation Implementation Follow-up evaluation Review and planning

TITLE: The topic seem to have a different understand as what’s in the context. The study is talking about the whole HH strategy of WHO while the focus is mostly on the first strategy “alcohol based handrub at point of contact. Again, study location should have been included in the topic to at least make it a bit clear. In that case, I advised you look and revise the topic.

- Thank you for this important point. You are right: in our paper we do not cover all components of the WHO strategy. Instead, we focus on (1) system change and (2) training - components where changes were most straightforward to measure. For your information: in the intervention: we also addressed (3) monitoring of practices and provision of feedback; (4) visual reminders in the workplace; and (5) creation of a safety climate within the institution.

We, therefore, clarified in the title “Mixed methods study evaluating the implementation of the WHO Hand Hygiene Strategy focusing on local production alcohol based handrub and training among health care workers in Faranah, Guinea”. We added further details to the method section p6 L5ff: “This paper focuses on the evaluation of the first two components of the multimodal WHO HH strategy: (1) system change, and (2) training and education [12]. But our intervention also addressed all other components: (3) monitoring and feedback, by immediate HH observations and direct feedback to the participants observed, (4) visual reminders in the workplace, by hanging up posters in the workplace and the distribution of flyers; and (5) creation of safety climate, by asking local staff for suggestions for improvement and patients for their personal opinion on HH.”

ABSTRACT

L18, 19 and 21 alcohol -based handrub was used without the abbreviation. However, in conclusion L3 the abbreviation (ABHR) was used. Please correct this, the abbreviation should be introduced in the first instance (L18) and only used thereafter. This process must be repeated in the main text so that you do not interchange alcohol-based handrub with ABHR.

- Thank you for spotting this mistake, abbreviation is now defined in L12 “(…) the relaunch of the local production of alcohol-based handrub (ABHR).” And used thereafter. In the main text the abbreviation is introduced in L10 and used thereafter.

INTRODUCTION

L3-4 “Healthcare-associated infections (HAI) are the most frequent adverse events worldwide.” This statement is very odd. Frequent adverse event following what? Hospital admission? Reference cited is old. From WHO fact sheet, there was specific case as to Healthcare Delivery Worldwide. So there should have been a focus on the refusal /omission of compliance or lack of knowledge of HH as a cause HAI.

- Thank you, we changed the statement and reference accordingly: “Healthcare-associated infections (HAI) are associated with a lack of awareness of hand hygiene (HH) standards, or the non-compliance with them [2].”.

L9 lack of running water in the facility, is that a reason for the research to focus mainly on only one of the 5 WHO HH strategies?

- As now clarified in method section p6 L5ff, our project addressed all components of the WHO HH strategy. Within the component “system change”, both sub-components 1) running water and 2) ABHR were addressed.

1)Running water: “Our project made an effort to improve the water supply, which over time improved indeed, albeit independently from our effort.” (p 5 L 9ff).

2) ABHR: To address the challenge of very unreliable ABHR supply within the hospital and the stated wish of the local staff for internal production of ABHR, our intervention focused on the local production of ABHR. However, we are well aware, that all WHO components complement and depend on each other. ABHR, therefore should not be seen as a substitute for reliable running water supply, but as one of the important HH strategy components to sustainably improve HH.

L21, 22, 23-24 show the research focus which is “to illuminate factors necessary for sustainability of local production of ABHR and to maintain HCW HH knowledge and compliance.” This clearly show that the key to the research is the first WHO HH strategy not the other four.

- Our paper focuses on the evaluation of two of the five WHO HH components, whereas all components were addressed in the intervention. For further detail please see method section p6 l5ff.

MATERIALS AND METHODS

Study Settings

L7 states that “the hospital lacks access to running water …” Is this the reason why Hand washing using soap, water and towel was not included, which is part 1b of the key WHO HH strategy?

- Apologies for not being clear enough in the paper: hand washing using soap, water and towel was included in the intervention, but was not the focus of this paper. Please also see answer to comment above.

Quantitative Study

L10-11 states that “The design, methods of the quantitative part and results of baseline and first follow-up of this study have been described in detail elsewhere.” Please indicate the where the details can be found? This is confusing!

- An in-text reference, pointing to the publication where these details can be found, directly follows the quoted statement. The reference is: Müller et al. 2020 doi: 10.1186/s13756-020-00723-8

L8-9 states that perception was compared with Wilcoxon rank-sum on phase III and IV while L10-13 stated that compliance was compared using Chi square WHO 5 Moment of HH strategy. Why are these different?

- Thanks for asking this question. We used the Wilcoxon rank sum test for the comparison of medians, the Chi2-test for the comparison of proportions. Compliance was summarised by proportions, knowledge and perception by medians, whereby perception was not compared between assessment phases. The section now reads: “Wilcoxon rank-sum tests were performed to compare median knowledge score at first and second follow-up with the baseline and to compare the HH perception of HCWs at first and second follow-up with each other. HCWs’ perception on the impact of project intervention was assessed in phase III and phase IV. HH compliance was observed directly and overtly without prior announcement and was calculated as the number of HH actions performed divided by the number of opportunities requiring HH according to the WHO “5 Moments of HH”. Chi2 tests was applied to compare proportions such as, compliance follow-ups to baseline and compliance at first to second follow-up.” P6 L17ff

Qualitative study

L2 “In-depth semi-structured interviews and focus group discussions.” Both introduced without abbreviations but abbreviations were used in L6 and L8 respectively.

- Thank you, we introduced the abbreviations in L12: “conducted a qualitative study with 22 in-depth semi-structured interviews (IDIs) and 6 focus group discussions (FGDs) with 4-8 participants” and took out the introduction of these abbreviations on page 9 L 10.

RESULTS

Hand Hygiene Knowledge

Qualitative

L8 states that one of the benefit form theoretical practical training was that “ABHR is faster (than handwashing) and does not cause dryness” Is this another reason for not considering hand washing as a key component of the strategy?

- Hand washing was considered a key component of the strategy, and the project undertook efforts to improve water supply at the hospital. From the next quote the reader can take that hand washing had its place in the strategy: “*thanks to PASQUALE I now know when to use ABHR, [and when to] wash my hands with soap or wear gloves*”. Still, one respondent mentioned as something he/she had learned that *“ABHR is faster [than handwashing] and does not cause dryness”*. We made no change to this section.

DISCUSSION

L7-8 states that “Nevertheless, knowledge at the second follow-up was still significantly higher than at baseline…” what is the baseline figure?

- Thank you, we added “(…)higher than at baseline in all professional groups (“Medical Doctor” 13.5, “Nurse” 11.5, “Midwife” 13.0, “Technician” 13.0) except for “Others”, consisting primarily of medical students and auxiliary nurses and presumably particularly affected by high staff turn-over [12].”

L19 states that “perception of the WHO multimodal HH strategy was positive throughout” This statement is not justified considering the fact that this study focuses mostly on just one aspect of the strategy

- Thank you, we specified in the method section that other aspects of the WHO multimodal HH strategy, such as workplace reminders, monitoring and feedback were also addressed in the intervention. We changed the statement in the discussion section to: “Perception of the WHO multimodal HH strategy was positive, especially on the component of local ABHR production and HH trainings for HCWs” p 17, L 20ff.

CONCLUSION

L20 states that “the WHO multimodal HH strategy may have a sustained positive effect on HCW compliance and knowledge in the FRH.” The positive effect suggested was mostly for 1a strategy: ABHR. Please review, perhaps describe the 5 point strategy first before focusing on only on point 1a.

- Thank you, we introduced the 5 components in the methods section and specified how every component was or was not addressed by the intervention, the evaluation and this paper. L 20 in the conclusion was clarified to: “The current study suggests that especially the local production of ABHR and HH training of the WHO multimodal HH strategy may have a sustained positive effect on HCW compliance and knowledge in the FRH”.
